# Evidence for Individual Face Discrimination in Non-Face Selective Areas of the Visual Cortex in Acquired Prosopagnosia

**DOI:** 10.1155/2008/561476

**Published:** 2008-04-11

**Authors:** Laurence Dricot, Bettina Sorger, Christine Schiltz, Rainer Goebel, Bruno Rossion

**Affiliations:** ^1^Department of NeurophysiologyUniversity of LouvainLouvain-la-NeuveBelgium; ^2^Department of Cognitive NeuroscienceMaastricht UniversityMaastrichtThe Netherlands; ^3^Maastricht Brain Imaging Center (M-BIC)MaastrichtThe Netherlands; ^4^Department of Cognitive DevelopmentUniversity of LouvainLouvain-la-NeuveBelgium

**Keywords:** Prosopagnosia, fMRI, fusiform gyrus, FFA, OFA, vLOC, adaptation

## Abstract

Two areas in the human occipito-temporal cortex respond preferentially to faces: ‘the fusiform face area’ (‘FFA’) and the ‘occipital face area’ (‘OFA’). However, it is unclear whether these areas have an exclusive role in processing faces, or if sub-maximal responses in other visual areas such as the lateral occipital complex (LOC) are also involved. To clarify this issue, we tested a brain-damaged patient (PS) presenting a face-selective impairment with functional magnetic resonance imaging (fMRI). The right hemisphere lesion of the prosoagnosic patient encompasses the ‘OFA’ but preserves the ‘FFA’ and LOC [14,16]. Using fMRI-adaptation, we found a larger response to different faces than repeated faces in the ventral part of the LOC both for normals and the patient, next to her right hemisphere lesion. This observation indicates that following prosopagnosia, areas that do not respond preferentially to faces such as the ventral part of the LOC (vLOC) may still be recruited to subtend residual perception of individual faces.

## 1. Introduction

The lateral occipital complex (LOC) plays a central role in human object recognition [13]. It is located anterior to retinotopic visual areas, extending both ventrally (vLOC) on the lateral bank of the fusiform gyrus and dorsally (dLOC) in two anatomically segregated subregions. Anterior to the vLOC, a region of the fusiform gyrus, the 'FFA' [11] responds more strongly to faces than to various non face stimuli. Larger responses to faces are also consistently observed in the 'occipital face area' ('OFA' [8]) generally posterior to, and partially overlapping with the vLOC. FMRI-adaptation [10] studies show a larger response in the LOC to novel objects than to repeated objects (e.g. [1]) and a correlation of that response with recognition performance (e.g. [9]). Similarly, fMRI-adaptation paradigms have shown that both the 'FFA' and 'OFA' are involved in the individual discrimination of faces (e.g. [8]). An unresolved issue is whether visual areas that do not respond preferentially to faces, such as the LOC, nevertheless contribute to the discrimination of members of that category.

Here we aimed to shed light on this issue by recording fMRI-adaptation in a brain-damaged patient who is no longer able to recognize and discriminate individual faces, i.e. prosopagnosia. The patient's ability to recognize nonface objects is remarkably preserved [14, 15]. Her prosopagnosia follows a dominant right hemispheric lesion in the inferior occipital cortex, which

ISSN 0953-4180/08/$17.00 © 2008 – IOS Press and the authors. All rights reserved damaged the territory of the right 'OFA'. However, the lesion spared the entire vLOC, as well as the right 'FFA' [14,16]; Fig. 1). The unique pattern of structurally damaged and intact tissue in this patient's brain allowed us to test whether areas that do not respond preferentially to faces, such as the vLOC, may still be recruited to subtend individual face discrimination.

## 2. Materials and methods

### 2.1. Subjects

The prosopagnosic patient PS has been already described in detail in previous studies [6,14–16]. PS is like normal subjects to discriminate faces from other objects but is impaired and slowed down to recognize faces at the individual level [15]. She does not present any difficulty in recognizing objects, even at the subordinate level [14,15]. A group of six control subjects (age range 25 to 35, 3 females) performed the same experiments.

### 2.2. Stimuli and procedures

In the 'FFA' localizer experiment, PS and controls viewed 8 blocks per run (36 s per block, two runs of 6 min 42 s) of alternating pictures of faces and objects, with 12s fixation between blocks. They performed a one-back identity task. 36 stimuli (4° of visual angle) were presented for 800 ms followed by a 200 ms blank screen during each block. Subjects were also scanned during an independent LOC localizer [16].

In the event-related fMRI experiment, subjects viewed three runs (8 min 57 s 500 ms per run) of 60 pairs of cropped and colored faces in frontal views in a delayed matching task. The first face was presented during 1000 ms following by a blank of 500 ms and thereafter by the second face of the pair for 1000 ms. Pairs were separated by a fixation cross during 5000, 6250 or 7500 ms.

MR images of brain activity were collected using a 3T head scanner with repeated single-shot echo-planar imaging: echo time (TE) = 50 ms, flip angle (FA) = 90°, matrix size = 64 × 64, field of view (FOV) = 224 × 224 mm, slice thickness = 3.5 mm. The other scan parameters were repetition time (TR) = 1500 ms, 24 slices, run time = 6 min 42 s for the 'FFA' localizer, TR = 2000 ms, 24 slices, run time = 5 min 20 s for the vLOC localizer and TR = 1250 ms, 21 slices, run time = 8 min 57 s 500 ms for the event-related face discrimination experiment. A whole brain three-dimensional (3D) T1-weighted anatomical data set (resolution = 1 mm3) was also acquired (TR = 7.92 ms, TE = 2.4 ms, FA =15°, matrix size = 256 × 256, FOV = 256 × 256 mm2, 176 slices, slice thickness = 1 mm, no gap, total scan time = 13 min and 43 s). fMRI signal in the different conditions was compared using BrainVoyager QX. Preprocessing consisted of a linear trend removal, a temporal high-pass filtering (>3 cycles per run) and a correction for interscan head movements. Data from the event-related experiment were also corrected for the difference between the scan times of the 21 slices. All volumes were spatially normalized [17]. Functional data were analyzed using multiple regression models consisting of predictors, which corresponded to the particular experimental conditions of each experiment [5]. An adaptation index allowing a comparison between PS and the control group was computed [(different-same)/(different + same)] using the beta weights of the two predictors of our event-related experiment (same faces and different faces conditions). fMRI signals averaged over each subject's ROIs were also extracted and percent signal change was computed using the baseline epochs as reference for each condition.

## 3. Results

The areas were defined in each subject individually by contrasting the percent signal change in response to faces as compared to pictures of common objects across the conjunction of two runs [run1(faces – objects) & run2 (faces – objects)] . For each subject, all contiguous voxels significant at $t > 5.59$ [one-tailed, p(Bonferroni corrected) < 0.002] in the right fusiform gyrus were considered as defining the 'FFA' (control subjects: 36 ± 4, −48 ± 7, −15 ± 4; mean cluster size: 708 voxels ± 516; PS: 35, −53, −20; 479 voxels) (Table 1). The right vLOC was defined by comparing common objects with the same stimuli scrambled, and identifying all contiguous significant voxels in the inferior occipital cortex of the right hemisphere (control subjects: 40 ± 4, −66 ± 9, −11 ± 4; 302 voxels ± 193; PS: 43, −64, −12; 632 voxels).

### 3.1. Event-related fMRI during face discrimination

Normal participants performed the discrimination task at ceiling (mean = 99.1% ± 0.74%) whereas PS's accuracy was at 86.2%. PS (1379 ms across conditions)

Table 1
Talairach locations, t-values and cluster sizes of the functionally defined regions of interests (right-sided 'FFA', 'OFA' and vLOC) defined in the localizer experiments for PS and the control subjects

| subject | region | Talairach coordinates | | | t | cluster size |
|---|---|---|---|---|---|---|
| | | x | y | z | | ($mm^3$) |
| PS | 'FFA' | 35 | −53 | −20 | 5.59 | 479 |
| | 'OFA' | | lesioned | | | |
| | vLOC | 43 | −64 | −12 | 6 | 632 |
| | 'FFA' | 39 | −44 | −16 | 8 | 526 |
| S1 | 'OFA' | 39 | −70 | −19 | 8 | 253 |
| | vLOC | 37 | −69 | −15 | 8.25 | 278 |
| S2 | 'FFA' | 37 | −41 | −16 | 5.59 | 1410 |
| | 'OFA' | 36 | −68 | −16 | 5.59 | 2545 |
| | vLOC | 38 | −73 | −9 | 7 | 245 |
| S3 | 'FFA' | 31 | −58 | −9 | 5.59 | 464 |
| | 'OFA' | 24 | −73 | −10 | 5.59 | 171 |
| | vLOC | 34 | −63 | −14 | 8.35 | 61 |
| S4 | 'FFA' | 39 | −49 | −20 | 10 | 654 |
| | 'OFA' | 35 | −67 | −18 | 10 | 64 |
| | vLOC | 43 | −68 | −9 | 8.25 | 233 |
| S5 | 'FFA' | 34 | −41 | −15 | 5.59 | 1795 |
| | 'OFA' | 28 | −79 | −7 | 5.59 | 153 |
| | vLOC | 41 | −71 | −6 | 6 | 567 |
| S6 | 'FFA' | 39 | −47 | −18 | 9.6 | 91 |
| | 'OFA' | 38 | −72 | −11 | 9.6 | 43 |
| | vLOC | 38 | −41 | −18 | 3.6 | 263* |
| S1–S6 | 'FFA' | 37 ± 3 | −47 ± 6 | −16 ± 4 | – | 823 ± 644 |
| S1–S6 | 'OFA' | 33 ± 6 | −72 ± 4 | −14 ± 5 | – | 538 ± 986 |
| S1–S6 | vLOC | 39 ± 3 | −64 ± 12 | −12 ± 5 | – | 277 ± 183 |

Remarks: *q (False Discovery Rate) < 0.05, otherwise: clusters defined at $p < 0.002$ (Bonferroni corrected for multiple comparisons).

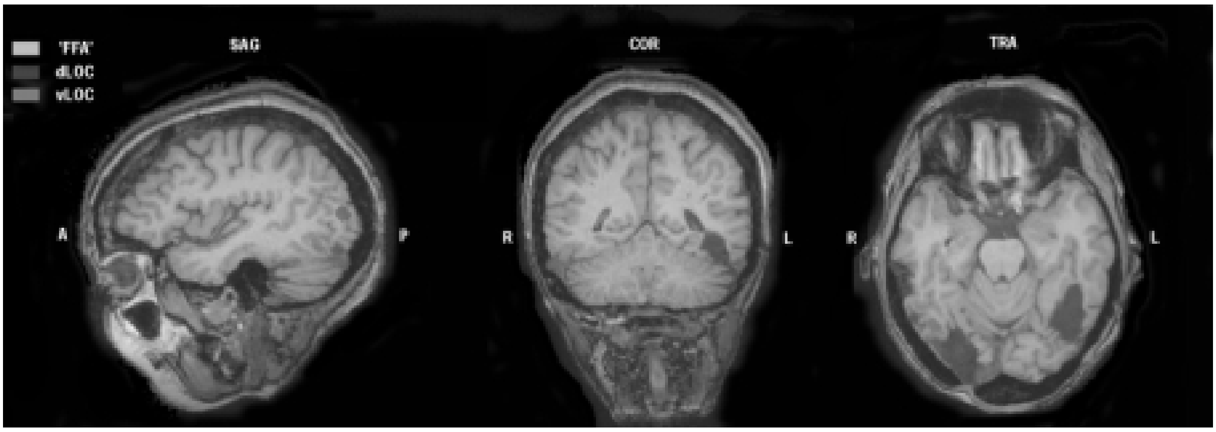

Fig. 1. Functional areas of the patient PS on brain slices. 'FFA': 'fusiform face area', area responding more to faces than objects in the right fusiform gyrus; dLOC and vLOC: dorsal and ventral part of the lateral occipital complex, area responding more to objects than scrambled objects.

was also slower ($t = 6.169, p < 0.000$) than controls (659 ms ± 113 ms). There were strong releases from adaptation (Fig. 2) in both the 'FFA' (random effect analysis: $p < 0.001$; individual p-values: ps < 0.048) and the vLOC (random effect analysis: $p < 0.012$; ps < 0.016) of the normal participants. In contrast, PS did not show release from adaptation to individual faces in the 'FFA' ($p = 0.46$) but a significant effect in the vLOC ($p < 0.00368$) only. When comparing PS's indices directly to those of the controls (Fig. 2), there was a significant difference in the 'FFA' ($t = -2.041$, $p < 0.048$; modified t-test [7]), but not in the vLOC ($t = 0.057$, $p = 0.478$), indicating that the magnitude of the effect was as large for PS and normal controls.

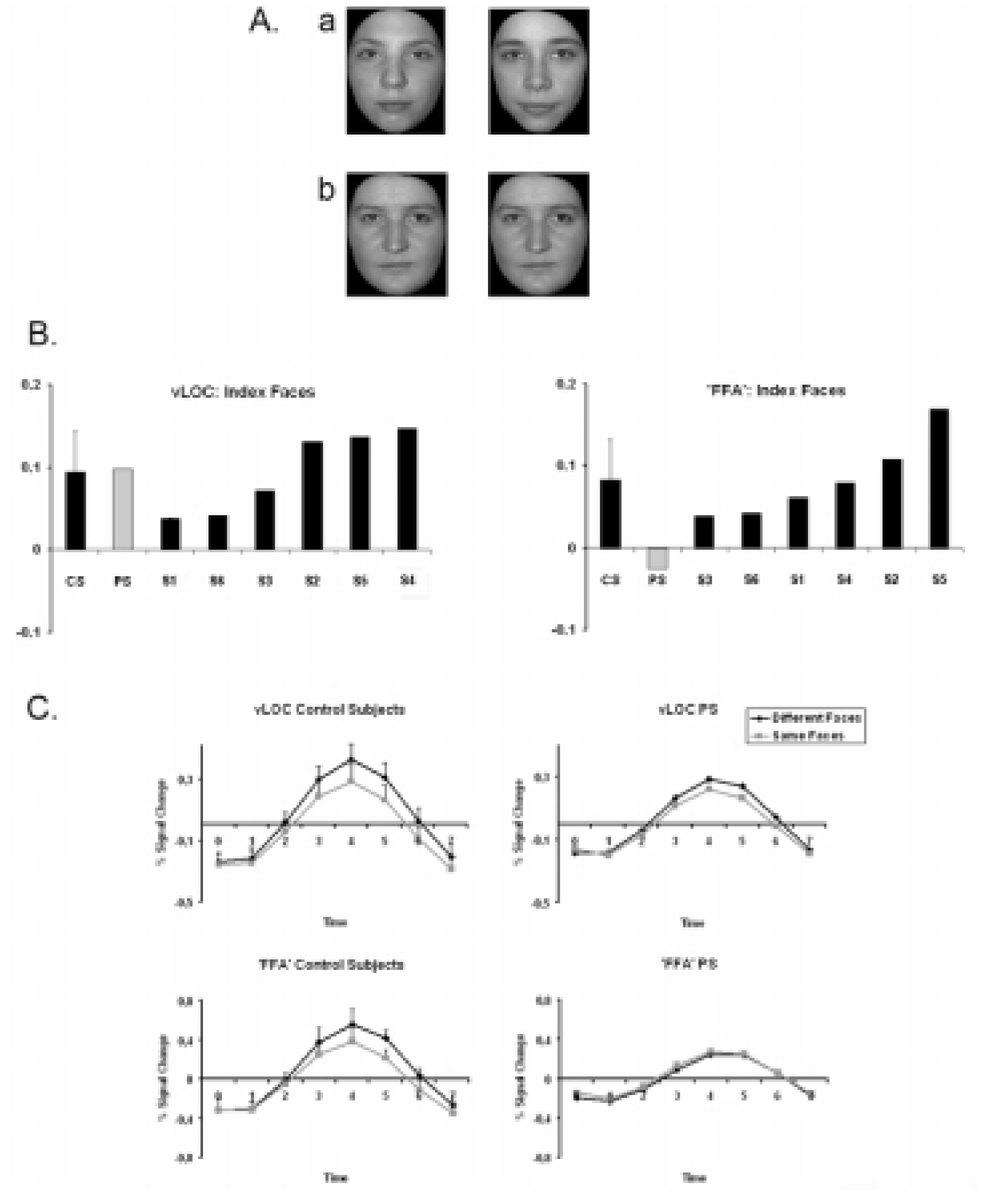

Fig. 2. Comparison between PS and the control subjects in the event-related fMRI-face discrimination. **A.** Two conditions were presented to PS and control subjects: second face different than the first (a) and second face identical to the first (b). All the faces were shown on frontal view presented in colour and sustained a size of roughly 4° of visual angle. The faces were cropped. **B.** Comparison between PS and the control subjects (CS): an Faces Index [(different–same)/(different + same)] was computed for PS's and each subject's vLOC and 'FFA' using the beta weights of the predictors used in the linear regression model. The Faces Index in the 'FFA' was significant for all subjects but PS contrary to her adaptation index in the vLOC which is significant and of identical magnitude to the control's index. **C.** PS's and control subjects' time-courses in the right-hemispheric vLOC and 'FFA'. PS did not show any evidence of a release from adaptation in the 'FFA', but normal effects in the vLOC.

## 4. Discussion

Despite her massive prosopagnosia, PS's performance in the active face discrimination task was at 86.2% but was slowed down relative to controls, who performed at ceiling. Such residual abilities are commonly observed in prosopagnosic patients, who may obtain relatively good scores at the Benton face matching tests [3] with unlimited time presentation (e.g. [12]). There is now strong evidence that these residual individual face discrimination and recognition abilities are not subtended by the 'FFA' of the patient, since this region does not show release from adaptation effects to identity, no matter the different procedures and stimuli used [15]; the present experiment. However, the present fMRI-adaptation experiment indicates that high-level visual areas that do not respond preferentially to faces, such as the vLOC, may subtend complementary visual processes to discriminate individual faces. The data strongly suggest that these processes are independent from processes taking place in areas responding maximally to faces ('FFA' and 'OFA') because there was no evidence of individual face discrimination in the latter regions: the 'OFA' is structurally damaged and the 'FFA' does not show release from adaptation to face identity. These observations suggest that there are multiple processes, with a certain degree of independence, which allow the extraction of an individual face representation in the normal brain. When the most efficient processes, requiring the 'FFA' and 'OFA' are unavailable, one may still rely on alternative processes in areas that do not mainly respond to faces (e.g. vLOC).

Moreover, the discriminative responses of facial identities observed in the vLOC of the patient and in the normal brain are insufficient to carry efficient face discrimination behaviour. Whereas the role of the 'OFA', and most probably the 'FFA' is critical for efficient discrimination of individual faces and recognition [2,4,14, 15] the vLOC appears to carry different and complementary functions that may or may not be necessary for face processing.
